# *Notes from the Field:* Cholera Outbreak — Zimbabwe, September 2018–March 2019

**DOI:** 10.15585/mmwr.mm6917a3

**Published:** 2020-05-01

**Authors:** Alison Winstead, Jonathan Strysko, Pryanka Relan, Erin E. Conners, Andrea L. Martinsen, Velma Lopez, Melissa Arons, Kudzai P.E. Masunda, Innocent Mukeredzi, John Manyara, Clemence Duri, Tapfumanei Mashe, Isaac Phiri, Marc Poncin, Nandini Sreenivasan, Rachael D. Aubert, Laurie Fuller, Shirish Balachandra, Eric Mintz, Portia Manangazira

**Affiliations:** ^1^Epidemic Intelligence Service, CDC; ^2^Division of Foodborne, Waterborne, and Environmental Diseases, National Center for Emerging and Zoonotic Infectious Diseases, CDC; ^3^Division of Global Health Protection, Center for Global Health, CDC; ^4^Division of Global HIV and TB, Center for Global Health, CDC; ^5^City of Harare Health Department, Zimbabwe; ^6^National Microbiology Reference Laboratory, Zimbabwe; ^7^Ministry of Health and Child Care, Zimbabwe; ^8^World Health Organization, Geneva, Switzerland; ^9^Global Immunization Division, Center for Global Health, CDC; ^10^Division of Global HIV and TB, Center for Global Health, CDC, Zimbabwe.

During September 5–6, 2018, a total of 52 patients in Harare, Zimbabwe, were hospitalized with suspected cholera, an acute bacterial infection characterized by watery diarrhea. Rapid diagnostic testing was positive for *Vibrio cholerae* O1, and on September 6, Zimbabwe’s Ministry of Health and Child Care (MOHCC) declared an outbreak of cholera. From September 4, 2018, (date of the first reported cases) through March 12, 2019, a total of 10,730 cases and 69 (0.64%) deaths were reported nationally from nine of Zimbabwe’s 10 provinces (Figure). Most cases (94%) were reported from Harare Province, the country’s largest province, with a population of approximately 2 million.

**FIGURE:**
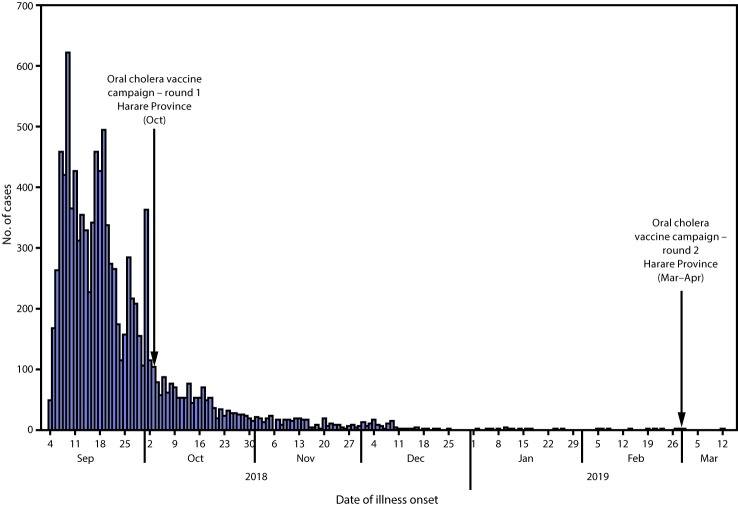
Suspected and confirmed cholera cases — Zimbabwe, September 2018–March 2019

Cholera outbreak response efforts were led by MOHCC in partnership with the City of Harare, the World Health Organization (WHO),* and many local and international organizations. Zimbabwe’s MOHCC activated its Inter-Agency Coordinating Committee on Health, which met regularly to coordinate response activities. Enhanced surveillance and reporting were encouraged nationally, and in Harare Province, supplementary surveillance trainings were provided by CDC to frontline medical staff members. In addition, approximately 200 health care workers received Integrated Disease Surveillance and Response training established by Zimbabwe, a comprehensive strategy for strengthening public health surveillance and response systems adopted by WHO African Region in 1998. This training-of-trainers effort was supported by MOHCC, WHO, and Africa Centres for Disease Control and Prevention. Cholera treatment centers were set up in affected areas in collaboration with Médecins Sans Frontières, and on-site case management trainings were conducted.

Laboratory testing at Zimbabwe’s National Microbiology Reference Laboratory confirmed *V. cholerae* O1 serotype Ogawa as the causative agent. Multiple organizations worked with the laboratory to provide supplies and training to enhance national and regional laboratory capacity. Antimicrobial susceptibility testing was confirmed at the National Institute for Communicable Diseases in South Africa.

MOHCC’s National Coordination Unit, with support from the United Nations Children’s Fund (UNICEF), initiated community-wide water, sanitation, and hygiene (WASH) interventions, including distributions of household water treatment products and water quality monitoring, within 1 week of the outbreak declaration. In late October, following the decrease in cases, more targeted interventions were introduced, including the use of integrated City of Harare and nongovernmental environmental health response teams that conducted case investigations, provided health education, distributed soap and household water treatment products to the index and surrounding households, and implemented point-of-collection chlorination at priority water points.

A 2-dose oral cholera vaccination campaign was conducted in Harare, beginning on October 3, 2018. Approximately 1.2 million doses administered by 1,750 health care workers completed the first round (administrative coverage = 86%) (Figure). During March–April 2019, a second round was conducted, with approximately 1.4 million doses administered by 1,900 health care workers (administrative coverage = 95%). The last reported cholera case occurred on March 12, 2019.

The first reported cholera case in Zimbabwe occurred in 1972, and in recent years, outbreaks have been reported almost annually. The largest outbreak recorded in Zimbabwe (and one of the largest ever in Africa) occurred during 2008–2009; 98,592 cases were reported, with 4,288 (4.3%) deaths (*1*). WHO advises that, with proper treatment, cholera case fatality should remain <1% (*2*); during the 2018–2019 outbreak, the case fatality rate was 0.64%, including deaths occurring within communities and at health facilities.

The timely declaration of this outbreak proved crucial to early response activities and resource mobilization. Prevention through improved WASH, community engagement, and cholera vaccination, as well as timely, integrated cholera outbreak detection and response activities are important to reducing the impact of cholera. Effective cholera outbreak response relies on collaboration among partners to systematically address the critical response pillars, including WASH, surveillance, laboratory testing, social mobilization, case management, and vaccination. Building local capacity through training remains a vital component of global health security, necessary to prevent, detect, and respond to infectious disease threats.
